# *Atractaspis aterrima* Toxins: The First Insight into the Molecular Evolution of Venom in Side-Stabbers

**DOI:** 10.3390/toxins5111948

**Published:** 2013-10-28

**Authors:** Yves Terrat, Kartik Sunagar, Bryan G. Fry, Timothy N. W. Jackson, Holger Scheib, Rudy Fourmy, Marion Verdenaud, Guillaume Blanchet, Agostinho Antunes, Frederic Ducancel

**Affiliations:** 1 Montréal University, Research Institute in Plant Biology, Montreal Botanical Garden, Montreal, Québec, Canada; 2 CIMAR/CIIMAR, Centro Interdisciplinar de Investigação Marinha e Ambiental, Universidade do Porto, Rua dos Bragas, 177, 4050-123 Porto, Portugal; 3 Departamento de Biologia, Faculdade de Ciências, Universidade do Porto, Rua do Campo Alegre, 4169-007, Porto, Portugal; 4 Venom Evolution Lab, School of Biological Sciences, The University of Queensland, St. Lucia, Queensland 4072, Australia; 5 Institute for Molecular Bioscience, University of Queensland, St Lucia, Queensland 4072, Australia; 6 Alpha Biotoxine, Montroeul-au-bois B-7911, Belgium; 7 CEA, IBiTec-S, Service de Pharmacologie et d’Immunoanalyse, Laboratoire d’Ingénierie des Anticorps pour la Santé, Gif-sur-Yvette, F-91191, France; 8 CEA, IBiTec-S, Service d’Ingénierie Moléculaire des Protéines, Laboratoire de Toxinologie Moléculaire et Biotechnologies, Gif-sur-Yvette, F-91191, France

**Keywords:** venom gland, transcriptome, *Atractaspis*, venomous snake

## Abstract

Although snake venoms have been the subject of intense research, primarily because of their tremendous potential as a bioresource for design and development of therapeutic compounds, some specific groups of snakes, such as the genus *Atractaspis*, have been completely neglected. To date only limited number of toxins, such as sarafotoxins have been well characterized from this lineage. In order to investigate the molecular diversity of venom from *Atractaspis aterrima*—the slender burrowing asp, we utilized a high-throughput transcriptomic approach completed with an original bioinformatics analysis pipeline. Surprisingly, we found that Sarafotoxins do not constitute the major ingredient of the transcriptomic cocktail; rather a large number of previously well-characterized snake venom-components were identified. Notably, we recovered a large diversity of three-finger toxins (3FTxs), which were found to have evolved under the significant influence of positive selection. From the normalized and non-normalized transcriptome libraries, we were able to evaluate the relative abundance of the different toxin groups, uncover rare transcripts, and gain new insight into the transcriptomic machinery. In addition to previously characterized toxin families, we were able to detect numerous highly-transcribed compounds that possess all the key features of venom-components and may constitute new classes of toxins.

## 1. Introduction

Snakes within the genus *Atractaspis* (family Lamprophiidae) represent one of three lineages that have independently evolved a sophisticated high-pressure, front-fanged venom delivery system; with the Elapidae and Viperidae constituting the other two lineages [1]. These oviparous snakes are largely fossorial and are distributed throughout sub-Saharan Africa with limited penetration into Israel and the southwestern part of the Arabian Peninsula [2]. The first species of *Atractaspis* was described in the mid-19th century, and, to date, eighteen different species are described [3]. From a morphological point of view, their venom system is unique since they bite with a single fang that projects sideways, allowing them to jab their prey with a closed mouth [4].

Little is known of the toxicity of the species of the genus as information is available on only a few taxa but common primarily effects are local swelling and necrosis. More dramatic manifestations have also been described as severe cardiotoxic effects and haemorrhagic activities [5,6]. As well as being morphologically unique, *Atractaspis* snakes are, to date, the only venomous species known to secrete sarafotoxins [3,7]. Sarafotoxins are a class of cardiotoxic peptides, ranging from 21–25 residues in length, which primarily induce coronary vasoconstriction [8,9,10]. These peptides are derived forms of endothelins, a class of vasoconstrictor peptides (21 amino acid residues) found in vertebrate vascular systems [2,11,12].

Prey subjugation is the primary function of *Atractaspis* venoms, but, like all snake venoms, they may also be utilized in a secondary defensive role. The toxin cocktail comprises of molecules that target various key regulatory pathways and a diverse array of protein families has been recruited into the myriad of animal venoms [13,14,15,16]. *Atractaspis* venoms have been poorly characterized as a whole and most prior studies have focused their attention on sarafotoxins [2,8,9,10,17]. Nonetheless, based on the phylogenetic placement of *Atractaspis*, as well as current knowledge of various snake venoms, we might expect a wide range of toxins or venom compounds to be secreted in their venom gland lumen. This could potentially include 3FTx (three finger toxin); acetylcholinesterase; AVIT, β-defensin; CRiSP; cystatin; C3/CVF (complement 3/cobra venom factor); epididymal secretory protein; hyaluronidase; kallikrein; kunitz; L-amino acid oxidase; lipocalin; lectin; natriuretic peptide; nerve growth factor; Type I phospholipase A2 (PLA2); Type IIE PLA2; phosphodiesterase; ribonuclease; renin aspartate protease; SVMP (snake venom metalloprotease); veficolin; vespryn; and waprin [1,15,16,18,19].

The present manuscript describes the first transcriptome analysis ever performed on *Atractaspis* snake venom glands (*Atractaspis aterrima*). Comparative analysis of normalized *versus* non-normalized libraries, using original tools including gene network, allows us to investigate the presence of a broad list of venom compounds. More generally, it contributes to a comprehensive characterization of both the toxin and non-toxin intracellular genes expressed in an actively transcribing snake venom gland.

## 2. Results

### 2.1. Sequencing and Assembly Statistics

As shown in Table 1, normalized and non-normalized libraries sequencing runs lead respectively to 724,119 and 581,370 reads of 344 and 315 mean bases length. In both cases, assembly using Newbler produced a similar number of contigs (69,975 and 57,962) covering about half of the reads.

**Table 1:** Statistics of 454 FLX sequencing and Newbler assembly.

	Normalized	Non-normalized
**Sequencing **		
Total Number of Reads	724,119	581,370
Total Number of Bases	249,123,133	183,358,305
Average Read Length	344	315
**Assembly Results**		
Number Assembled	427,470	266,199
Number tooshort	27,744	0
**Sum of Large Contigs (>1 Kb)**		
Total number of reads	86,119	35,356
Number of Large Contigs	2197	265
Total number of bases	2,914,941	344,247
**Sum of All Contigs **		
Total number of reads	427,470	266,199
Number of All Contigs	69,975	57,962
Total number of bases	35,504,970	22,851,131

In the present study, non-assembled reads were not used for further analysis, and were not considered for prediction of new toxin compounds, but this major pool of single reads could be of great interest for future investigation. This also shows that despite deep sequencing and as mentioned in previous studies [20], the function of a significant part of the transcriptome is still unknown. Comparison of the two data sets (Supplementary Figure 1) shows that it is necessary to annotate only 1105 contigs to access 80% of the non-normalized dataset *versus* 13,221 contigs for the normalized one. On one hand, the normalized library gives access to weakly express transcripts; on the other, the use of a non-normalized library is a more effective way to describe the general transcriptional activity of the venom gland thanks to the recovery of a smaller number of transcripts. This perfectly illustrates the pros and cons of each approach.

### 2.2. Functional Annotation of *Atractaspis aterrima* Venom Gland Transcriptome

**Figure 1:**
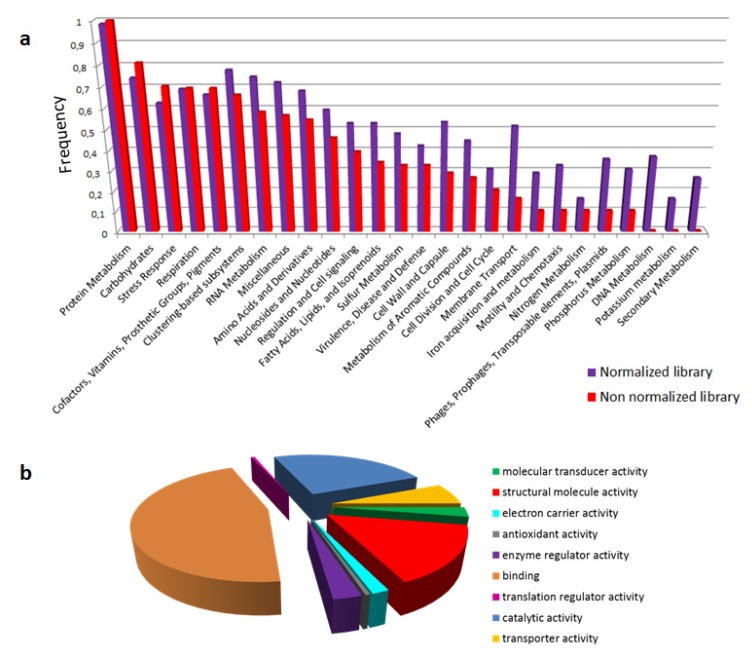
Functional annotation of *Atractaspis* transcriptomes. (**a**) Differences of subsystem’s annotation of reads between Normalized and Non-Normalized libraries. (**b**) Gene Ontology classification of reads covering 80% of assembled contigs (Non-Normalized library).

The overall analysis of the transcriptome based on subsystems revealed the prevalence of predicted functional categories related to protein synthesis and more generally to common intracellular activities (Figure 1a).

Such a result has already been observed for venom gland transcriptomes, and is consistent with the very active nature of these tissues [21,22]. It is noteworthy to mention that numerous transposable elements were also detected. Whether these genetic entities play a role in venom function is a question that is yet to be addressed. After focusing on sequences representing 80% of the transcripts in the non-normalized library we observed that functional gene ontology categories cover most activities associated with toxins themselves (Figure 1b). Thus, about half of the annotated sequences exhibit binding activity, and other major functions predicted include catalytic activities and structuring of molecules. For putative functional categories related to biological processes (data not shown), we note that the most abundant functions are related to general cellular activity, metabolic processes, and regulatory mechanisms. This underscores, more broadly, the intense metabolic activity of the venom gland.

### 2.3. Analysis of Toxin Transcripts

**Figure 2:**
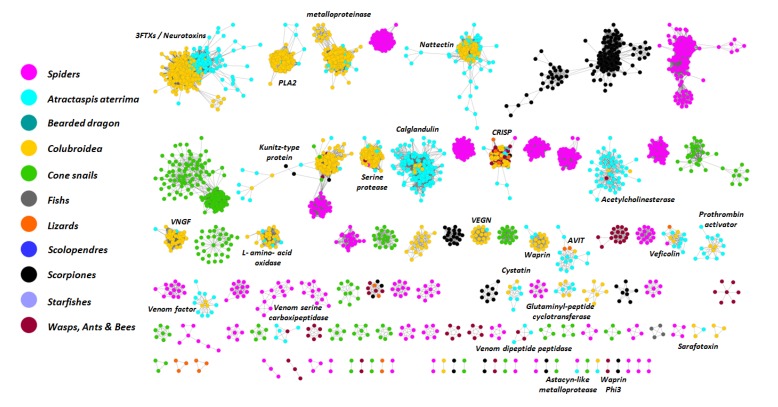
Network analysis of putative toxins. The network includes 6036 non redundant Toxins or associated venom protein classified by Uniprot (ToxProtDb) and 637 partial & full-length putative toxins from the present study. Minimal *e*-value for edge connexion is set to 1E^−10^.

Network based annotation of toxins illustrating the diversity of previously characterized toxins in the venom cocktail is represented in Figure 2.

Twenty-four different groups of venom gland compounds have been identified. In this graphical representation of shared similarities of toxins, the nodes (sequences) in the network are linked by edges. The closer the edges, the higher the shared similarity. Building a gene network allows sequences to be grouped into connected components based on their shared similarities. We are, thus, able to identify a broad spectrum of molecules in *A. aterrima* venom transcripts. Among them, most (16 of 24) venom components match toxin classes already identified in various groups of snakes (Table 2).

**Table 2:** List of expressed snake-related toxins in *A. aterrima* venom gland.

Toxin family	Isoform(s)	Remarkable feature
**Three finger toxin**	13	Large diversity
**Kunitz type/TFPI**	3	Original signal peptide/one Kunitz type domain
**AVIT**	3	Distantly related to dendroapsis and varanus AVITs
**Choline esterase**	6	Original signal peptide
**Crotamine**	1	Conserved signal peptide (*Crotalus* genus), conserved cysteine pattern but low similarity through mature peptide
**Metaloproteinase disintegrin (ADAM)**	1	Truncated sequence
**C-type lectin**	4	Two distinct groups
**Waprin**	6	None
**Kallikrein / Serine protease**	2	None
**CRISP**	1	Highly similar to Latisemin toxin from *Laticauda semifasciata*
**Venom nerve Growth Factor (VNGF)**	1	Partial sequence nearly identical to viperidae’s VNGFs
**Lipocalin**	3	Two different groups. Major compound of the venom gland’s transcriptome
**PLA2**	1	Partial sequence. Highly similar to phospholipase A2 type II from *Leioheterodon madagascariensis*
**Cystacin**	4	Partial sequences highly similar to *Crotalus adamanteus* toxins but lack signal peptide
**Sarafotoxin**	2	Partial sequence. Matching only two reads from the normalized library
**Calglandulines**	1	95% identical to the Elipadae *Austrelaps superbus* caglandulin sequence

For some toxin types, we were able to detect a large number of isoforms which may interact with different targets and therefore could cover a wide range of activities and mode of action. For instance, 13 full-length 3FTx sequences were identified in this transcriptome. Similarly, the lectin sequences were highly diverse relative to previously identified toxins as indicated by distant ramifications on the given connected components. An intriguing point of this study is that only two incomplete reads encoding sarafotoxin precursors were recovered, suggesting that sarafotoxins do not constitute the main component of the venom cocktail at the transcriptomic level. In both reads, the original poly-cistronic organization found in sarafotoxin-percursors of *A. engaddensis* and *A. microlepidota* was observed [8,9]. Nonetheless, inferred mature sarafotoxin sequences represent two new long-SRTX isoforms of 24 amino acid residues. One of the transcripts includes a full length mature SRTX sequence and thus constitutes the first SRTX sequence ever described in *A. aterrima*. This primary sequence combines the 21 first amino acid residues attributed to bibrotoxin from *Atractaspis bibronii* [23] with the C-terminal extension “DEP” found in all long-SRTXs of *Atractaspis microlepidota* [9].

Beyond these intensively studied families of snake toxins, another intriguing recovery was that of a contig matching an arachnid astacin-like metalloprotease (Supplementary Figure 2). While numerous convergent recruitment processes have been recently highlighted among venomous animals [13,14] this constitutes the first report of this toxin type in the snake venom arsenal.

### 2.4. Molecular Evolution of *Atractaspis aterrima* Three-Finger Toxins

**Figure 3:**
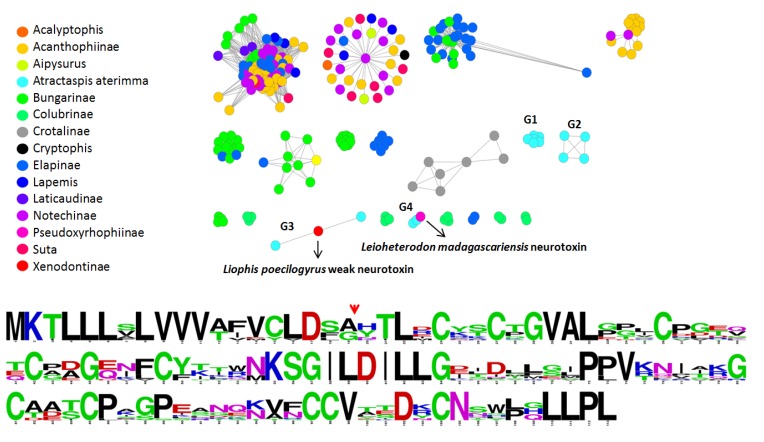
gene network analysis of snake’s 3FTXs and *A. aterrima* 3FTXs consensus sequence. Minimal e-value for edge connexion is set to 1E-20.

Since a large number of three-finger toxins (3FTxs) were sequenced in this study, the first time this toxin type has ever been recovered from a species of *Atractaspis*, it provided us with an opportunity to assess the molecular evolution of 3FTxs in this species. The 13 isoforms identified (consensus sequence presented in Supplementary Figure 3) all contained the ten cysteines of plesiotypic forms of 3FTx [16,18,24,25]. That the *Atractaspis* sequences are not monophyletic (Data not shown) is consistent with the early recruitment of this toxin type into the snake venom arsenal [16,18,25]. To complement the phylogenetic analyses, we chose a gene-network-based clustering approach (Figure 3).

Consistent with the phylogeny, the network analyses at a 1E^−20^ *e*-value threshold split the *A. aterrima* 3FTxs into four different groups (G1 to G4). One of the isoforms is not associated with any groups at this *e*-value threshold and is consequently classified as a singleton and does not appear in the graphical representation. It is noteworthy that none of the *A. aterrima* isoforms were found in any of the larger 3FTx groups.

**Figure 4:**
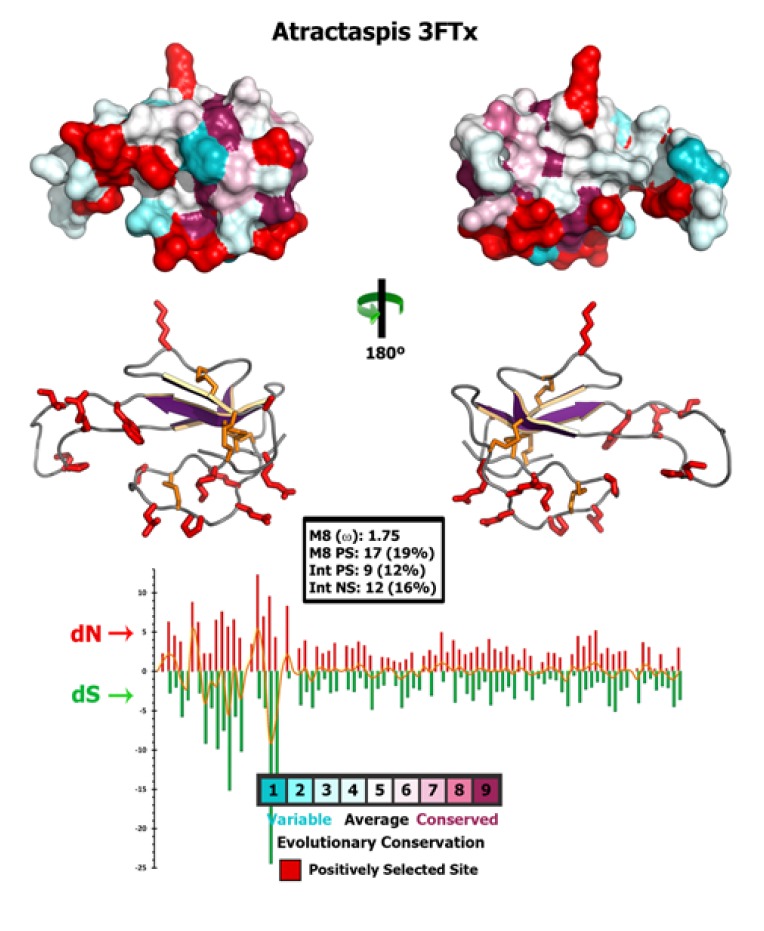
Three-dimensional homology model of *Atractaspis* 3FTx, depicting the locations of positively selected sites (shown in red) detected by site-model 8. The omega value and the number of positively selected sites (Model 8, PP ≥ 0.95, depicting the locations of poach) are also indicated.

Selection assessment of *Atractaspis* 3FTxs revealed the rapid evolution of these toxins under the influence of positive selection (Figure 4 and Supplementary Table 1).

Site-model 8 computed a *ω* value of 1.75 for these toxins, while the BEB approach implemented in this model identified as many 17 positively selected sites (39% of sites) (Supplementary Table 1). MEME identified 8 codon positions in *Atractaspis* 3FTx as experiencing episodic bursts of adaptive selection pressure (Supplementary Table 1), while the branch-site REL test detected as many as four branches as evolving under episodic diversifying selection (Supplementary Figure 4A). Evolutionary fingerprint analyses revealed a large proportion of rapidly diversifying sites in these toxins (Supplementary Figure 4B). A bayesian method was also employed to identify sites under ervasive diversifying and purifying selection pressures (Supplementary Table 1). Thus, various selection assessments highlighted the rapid diversification of *Atractaspis* 3FTx, indicating that they are possibly involved in an evolutionary arms race scenario with their prey, and detected a large number of sites as evolving under the influence of positive Darwinian selection (Figure 4 and Supplementary Table 1).

### 2.5. Analysis of Highly Expressed Transcripts and Detection of Unknown Proteins

**Figure 5:**
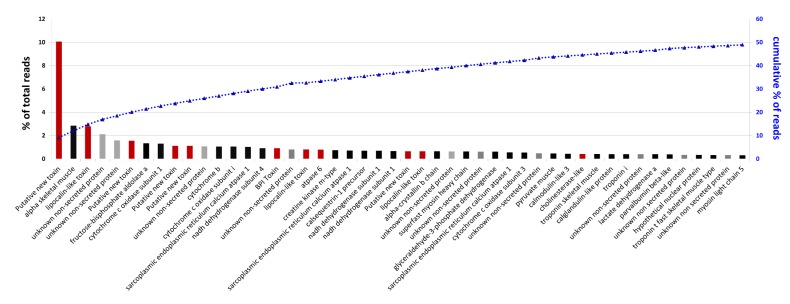
Annotation of the most abundant transcripts. Transcripts are sorted according to their abundance. In red are putative toxins, in grey are protein of unknown function and in black sequences that do not match these two categories.

We focused our attention on the 57 most abundant gene transcripts (Figure 5), constituting more than 50% of reads.

Surprisingly, we could not retrieve similar sequences from the GenBank database for the most abundant transcript recovered in this study, suggesting that this may be a new toxin type. SignalP program predicted a putative signal peptide for these transcripts, suggesting that this product is likely to be expressed in the venom glands. The mature sequence is 173 amino acids in length and includes two highly conserved cysteine residues. We were able to uncover at least 10 different isoforms from 34 full-length sequences. If similarly secreted, the high level of expression of this transcript in the venom gland suggests a “toxic” role for this new compound. However, functional assessments are required to confirm the secretion and determine the biological activities of these putative toxin transcripts. Using transcript abundance ranking, we were also able to identify three other “putative toxin” genes. Consensus sequences are provided in Supplementary Figure 5. Indeed, these new compounds (designated as #6, #9, #10, and #25) make up the vast majority of toxin transcripts recovered in this study. These results are remarkable and highlight the fact that even heavily studied venomous lineages like snakes can be a potential source of novel biochemical compounds, which may have applications as investigational ligands or in in drug design, and may be of significance in antivenom production.

Aside from these putative novel toxins, we were able to identify the primary functions of the most abundant transcripts. As described in a previous transcriptomic venom gland study [21], the major transcripts are associated with the cytoskeleton and protein synthesis. Among the transcripts of known toxin types, lipocalins are the most abundant. The toxic function of these proteins remains ambiguous, however, but lipocalins found in *Atractaspis atterima* could act as anticoagulant proteins [1]. Phylogenetic analysis of these sequences (Supplementary Figure 6) shows that two different groups have potentially been recruited into the *A. aterrima* venom cocktail. The first group is related to vertebrate (*Rana*, *Xenopus*, *Bufo*) lipocalins with non-toxin function—lipocalin G1 isoform from *A. aterrima* belongs to this group. Lipocalin G1 is highly expressed in the venom gland (rank 18, 0.8% of reads). The second and larger group of lipocalins includes two different isoforms named G2a and G2b (consensus sequences given in Supplementary Figure 5) related to snake lipocalins (Colubridae and Viperidae). They are also highly expressed in the transcriptome (rank 3 and 27 of the most abundant transcripts, 2.78% of total reads). These results, if confirmed by proteomic investigation, highlight the fact that the diversity of venom gland compounds is huge, and even in deeply studied groups as snakes, remains poorly understood.

## 3. Experimental Section

### 3.1. Snake Venom Gland cDNA Synthesis and Sequencing

The two venom glands were dissected and immediately frozen in liquid nitrogen and stored at −80 °C from a unique male specimen of *Atractaspis aterrima* collected alive in Tanzania and kindly provided by *Alpha Biotoxine* Company. The two non-elongate venom glands were later ground under liquid nitrogen. From the tissue powder, total RNAs were isolated using the mirVanamiRNA isolation kit (Ambion). The total RNA fraction was examined by capillary electrophoresis using Bioanalyzer (Agilent). The total RNA poly(A) was split in two samples for the construction of normalized and non-normalized libraries. First-strand cDNA synthesis was primed with a N6 randomized primer. Then 454 adapters A and B were ligated to the 5' and 3' ends of the cDNA. The cDNA was finally amplified with PCR (21 cycles) using a proof reading enzyme. For Titanium sequencing the cDNA in the size range of 500–700 bp was eluted from a preparative agarose gel. From this RNA pool, we generated a normalized and non-normalized library using standard protocols.

### 3.2. Bioinformatic Processing of the 454 Reads and Annotation of the Dataset

Sequences were trimmed to remove adapters and low quality regions. Reads were assembled using Newbler 2.3 (454 Life Science). An identity threshold of 98% was chosen for contig assembly with a minimal overlap of 40 bp. Open reading frames were predicted using GeneMark.hmm-E [26] and matching contigs and singletons of at least 60 amino acids in length were further annotated by similarity search on the NR database of GenBank, specifying an *e*-value cut-off of 1E−05. Snake venom gland-specific transcripts were then selected from best-BLAST hit descriptions using a list of keywords of all known toxin protein families described so far [18,27] in a broad range of venomous taxonomic groups. As most toxin sequences originate from duplication events of common cellular genes, retrieving toxin sequence by simple key-word search is highly prone to a false positive detection. To avoid this major pitfall, we trimmed putative toxins from the original list. We choose to select hits showing a best bidirectional BLAST hit on NR database. To analyze the diversity of the toxin repertory, stringent parameters were applied as following: exclusion of (i) truncated sequences, (ii) sequences with ambiguous positions, and (iii) sequences lacking a signal peptide after a SignalP software screening [28]. Nonetheless, we applied less stringent parameters for weakly expressed toxin families as we were sometimes unable to identify full-length sequences (see below). Information and datasets resultant from this project can be accessed from DDBJ/EMBL/GenBank under following accession numbers: Bioproject PRJNA210890, Biosample SAMN02230181, Sequence Read Archive (SRA) SRR931902 and Transcriptomic Shotgun Assembly SUB296805 and SUB291519.

### 3.3. Functional Annotation of Contigs

To characterize the major transcripts of the venom gland transcriptome, we have chosen to describe the most abundant transcripts representing 80% of the dataset using reads from non-normalized library. Although the relationship between the number of transcripts and the corresponding amount of protein expressed is not always linear, analysis of transcripts covering 80% of reads assembled into contigs can nevertheless highlight the key molecular components of venom production as well as the main compounds of the venom cocktail. The distribution of functional categories for COGs, KOs, NOGs, and Subsystems at the highest level supported by these functional hierarchies was processed after the BLAST step against NR protein database.

### 3.4. Prediction of Non-Matching Sequences

**Figure 6:**
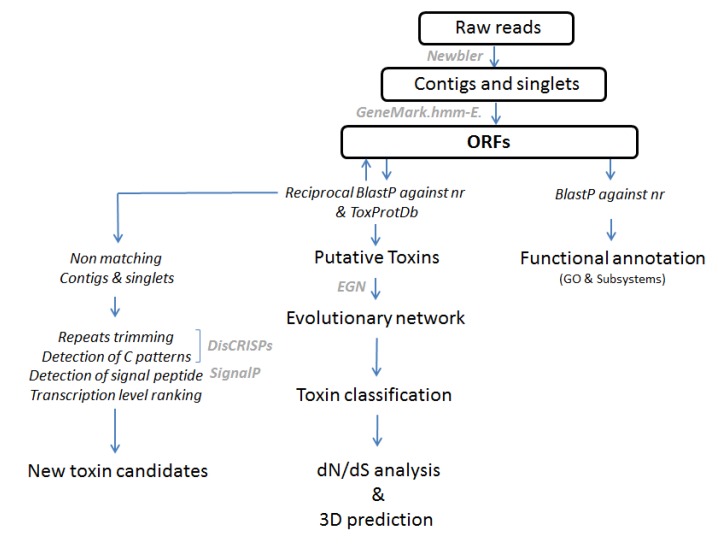
EST processing workflow.

Several studies have shown that transcriptomes include a large number of uncharacterized sequences whose function is still unknown. These sequences can cover several categories of transcripts including mainly non-coding RNAs [20]. We were particularly interested in the presence of transcripts which do not show strong similarities (*e*-value threshold set to 1E-05) with proteins or sequences already identified. For this, we combined the use of homemade software, which analyzes BLAST results, Signal P outputs and an optional detection of cysteine patterns. In the case of a venom gland, the detection of a transcript corresponding to a secreted protein suggests that the protein is most likely a toxin as the common core of the vertebrate genome is relatively well characterized. We completed these initial criterions by using the transcript levels associated with each of the new candidates to select the most relevant ones. The general EST processing workflow is described in detail in Figure 6.

### 3.5. Diversity Analysis Using Evolutionary Networks

Phylogenetic analysis of toxins remains the gold standard to estimate sequence diversity and evolutionary processes. Unfortunately such analysis can lead to unresolved topologies for the following reasons: (1) the toxins are mainly small peptides, and (2) they are often very diverse. Consequently, alignments produced for a given family are generally of low quality and the number of positions available to produce informative phylogenetic analysis supported is insufficient. This gives rise to the production of non-informative topologies. In order to the nature of these sequences, we chose to adopt a network analysis. To form the data set, we imported the curated sequences of toxins available on Uniprot (named ToxProtDB, [29]) and we produced a non-redundant data set using cd-hit clustering software [30]. These sequences were blasted on predicted ORFs to retrieve *Atractaspis aterrima* putative toxins. After being assured by a reciprocal blast on the NR Genbank database that these candidates were indeed potential toxins, we analyzed the full dataset following a standard protocol [31]. Networks were further visualized using Cytoscape [32].

### 3.6. Selection Analyses

The influence of natural selection on *Atractaspis* 3FTx was evaluated using maximum-likelihood models [33,34] implemented in CODEML of the PAML [35]. We employed site-specific models that estimate positive selection statistically as a non-synonymous-to-synonymous nucleotide-substitution rate ratio (*ω*) significantly greater than 1. We compared likelihood values for three pairs of models with different assumed *ω* distributions as no *a priori* expectation exists for the same: M0 (constant *ω* rates across all sites) *versus* M3 (allows the *ω* to vary across sites within “*n*” discrete categories, *n* ≥ 3); M1a (a model of neutral evolution), where all sites are assumed to be either under negative (*ω* < 1) or neutral selection (*ω* = 1), *versus* M2a (a model of positive selection), which in addition to the site classes mentioned for M1a, assumes a third category of sites; sites with *ω* > 1 (positive selection) and M7 (Beta) *versus* M8 (Beta and *ω*), and models that mirror the evolutionary constraints of M1 and M2 but assume that *ω* values are drawn from a beta distribution [36]. Only if the alternative models (M3, M2a, and M8: allow sites with *ω* > 1) show a better fit in Likelihood Ratio Test (LRT) relative to their null models (M0, M1a, and M7: do not show allow sites *ω* > 1), are their results considered significant. LRT is estimated as twice the difference in maximum likelihood values between nested models and compared with the χ^2^ distribution with the appropriate degree of freedom—the difference in the number of parameters between the two models. The Bayes empirical Bayes (BEB) approach [37] was used to identify codon sites under positive selection by calculating the posterior probabilities that a particular amino acid belongs to a given selection class (neutral, conserved or highly variable). Sites with greater posterior probability (PP ≥ 95%) of belonging to the “*ω* > 1 class” were inferred to be positively selected.

In addition, Fast, Unconstrained Bayesian Approximation (FUBAR) approach implemented in HyPhy package [38,39] was employed to provide additional support to the aforementioned analyses and to detect sites evolving under pervasive diversifying and purifying selection pressures. We also employed Mixed Effects Model Evolution (MEME) [39] to detect sites evolving under the influence of episodic diversifying selection. To clearly depict the pro-portion of sites under different regimes of selection, an evolutionary fingerprint analysis was carried out using the ESD algorithm implemented in datamonkey [40]. We also employed branch-site REL (BSR) test [41] implemented in HyPhy to identify lineages diversifying under the influence of episodic selection pressure.

### 3.7. Structural Analyses

To depict the influence of natural selection pressures on the evolution of *Atractaspis* 3FTx, we mapped the sites under positive selection on the homology model created using Phyre 2 webserver [42]. Pymol 1.3 [43] was used to visualize and generate the images of homology models. Consurf webserver [44] was used for mapping the evolutionary selection pressures on the three-dimensional homology models.

## 4. Conclusion

This study illustrates the potential for high throughput sequencing technologies in unearthing novel biochemical components, even from intensively studied venomous lineages. Such novel toxins are of great interest to drug design and development research, as well as in antivenom production. This study not only highlights the importance of gene-network analyses, but also the fact that similarity-based or assay-guided methods could fail to identify major components of a transcriptome. We show that sarafotoxins do not constitute the major ingredient of the *Atractaspis aterrima* venom cocktail at the transcriptomic level. This surprising result could be a result of the dramatic intraspecific variations of the venom cocktails. Moreover, using a combined transcriptomic and proteomic study [45], it was recently demonstrated that the ultimate venom composition of an animal is influenced by transcriptional and translational mechanisms that may be more complex than previously appreciated.

Of the novel toxins we identified, the identification of diverse 3FTx forms is particularly notable. This toxin type has diversified explosively in elapid snake venoms [24] as well as in various families of non-front-fanged snakes, Colubridae in particular [18,25]. As this toxin type was recruited at the base of the snake radiation [16], its presence in *Atractaspis* venom is not surprising. However, considering the molecular diversity present, these novel versions may prove useful as investigational ligands or even substrates for use in drug design and development.

In addition, of note are the transcripts encoding for enzymes similar to the arachnid astacin-like metalloprotease protein. The spider toxins are zinc metalloproteases that causes de-adhesion of endothelial cells from cell cultures, and also degradation of fibronectin, fibrinogen and gelatin *in vitro* [46]. Their role in venom is not fully understood but they might act as a spreading factor facilitating diffusion of other venom toxins. Alternatively, they might be involved in the proteolytic processing of other venom toxins or may play a role in predigestion of prey. The partial sequence identified in our study is also highly similar to fish hatching enzymes [47] but it is not related to any previously characterized reptilian gene. Comparing the partial sequence identified in our specimen with its closest relatives (fishes and spiders) demonstrates a nearly perfect shared sequence identity with the sequence of the *Fundulus heteroclitus* hatching enzyme (Supplementary Figure 2). At this stage, we cannot exclude an endogenous function for this protein in the venom gland, but it may also be present as the result of a recent recruitment event.

This study highlights the usefulness of new annotation tools for fast and accurate annotation of transcripts. To our knowledge, this study is the first time a gene network has been used for toxin annotation. The major advantage of this method is that it allows quick annotation and comparison of the venom cocktail to previously characterized toxins via a graphical view. Moreover, it complements traditional methods of studying toxin evolution through use of phylogenetic trees and multi-domain protein incongruences. This work should obviously be completed by a thorough functional investigation of the toxin types identified. In particular, the four new toxin families are of great interest as they are major components of the venom cocktail. This study also highlights that small taxonomic groups are of great interest in the field of toxin discovery and that particular effort and attention has to be given to the investigation of such rarities 

## Supplementary Files

Supplementary File 1 Supplementary (PDF, 1628 KB)
