# Supplementary material for: Atractaspis aterrima Toxins: The First Insight into the Molecular Evolution of Venom in Side-Stabbers
Source: Toxins (Basel). 2013 Oct 28;5(11):1948–64. doi: 10.3390/toxins5111948 (PMC3847709; doi:10.3390/toxins5111948)
Supplement: Supplementary File 1 — Supplementary (PDF, 1628 KB) [file toxins-05-01948-s001.pdf]

Supp. Fig. 1: cumulative coverage of Contigs (a) and reads (b) of normalized (red) and non-Normalized (blue) libraries

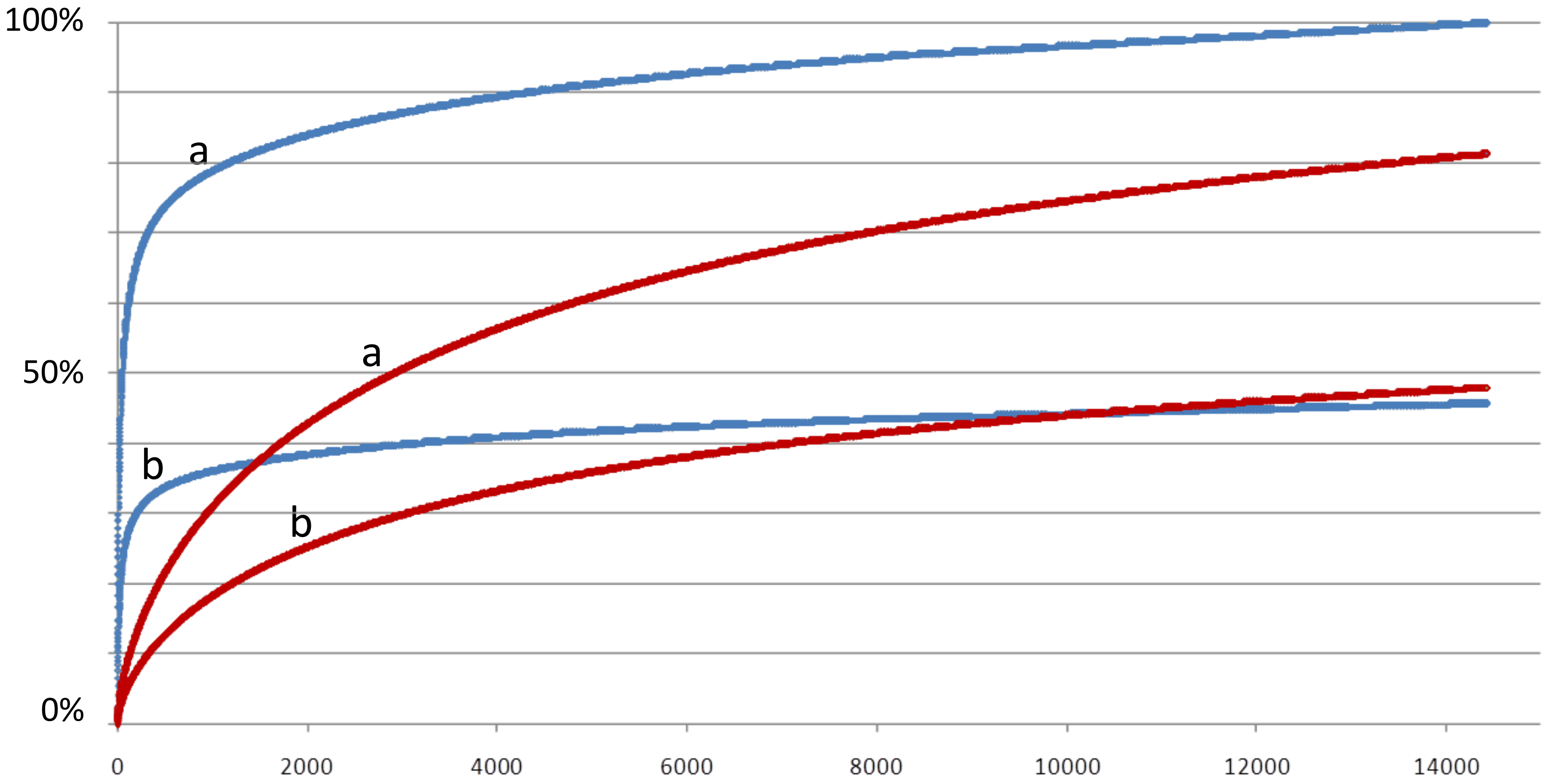

Supp. Fig. 2 : proteic alignment of Atractaspis astacin-like metalloproteases and related fishe's hatching enzymes

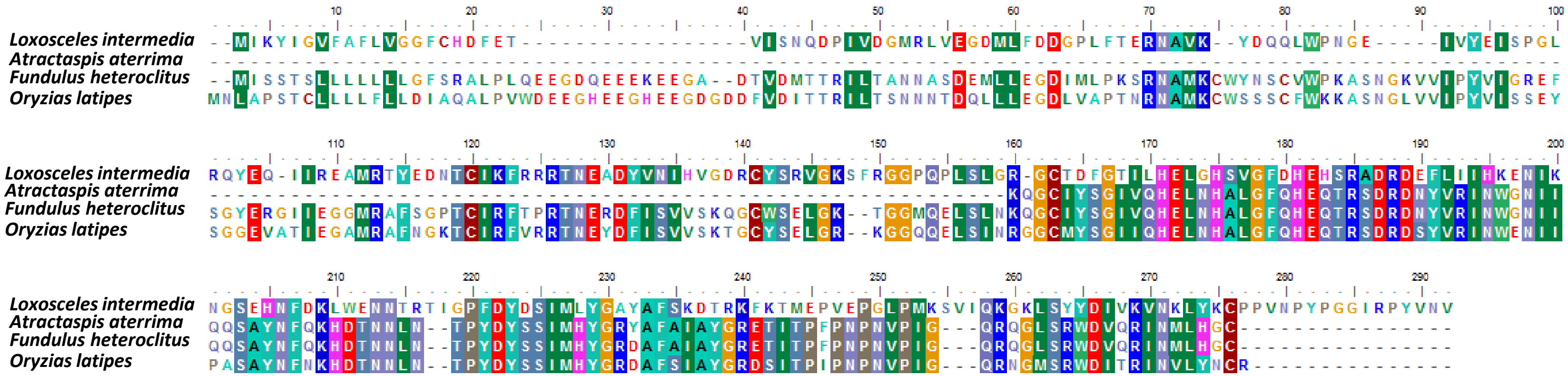

Supp. Fig 3 : proteic alignment of 3FTXs isoforms
Treshold for identity/similarity shading is set to 60%.

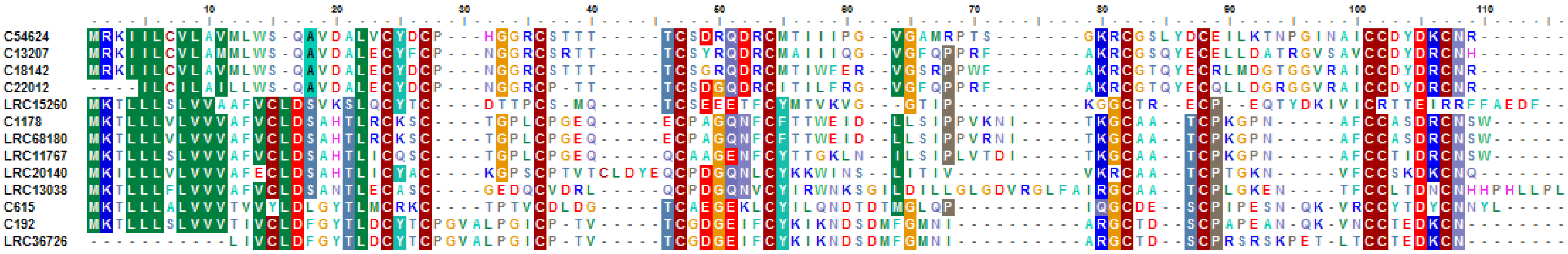

Supp. Fig. 4: Molecular evolution analysis of Atractaspis three-finger toxins

A : The hue of each colour indicates strength of selection, with primary red corresponding to ω > 5, primary blue to ω = 0 and grey to ω=1. The width of each colour component represents the proportion of sites in the corresponding class. Thicker branches have been classified as undergoing episodic diversifying selection (indicated by arrows) by the sequential likelihood ratio test at corrected p ≤ 0.05.

B : Estimates of the distribution of synonymous (alpha) and non-synonymous (beta) substitution rates inferred for Atractaspis three-finger toxin (3FTX). The ellipses reflect a Gaussian-approximated variance in each individual rate estimate, and colored pixels show the density of the posterior sample of the distribution for a given rate. The diagonal line represents the idealized neutral evolution regime (ω = 1), points above and below the line correspond to positive selection (ω>1) and negative selection (ω<1), respectively.

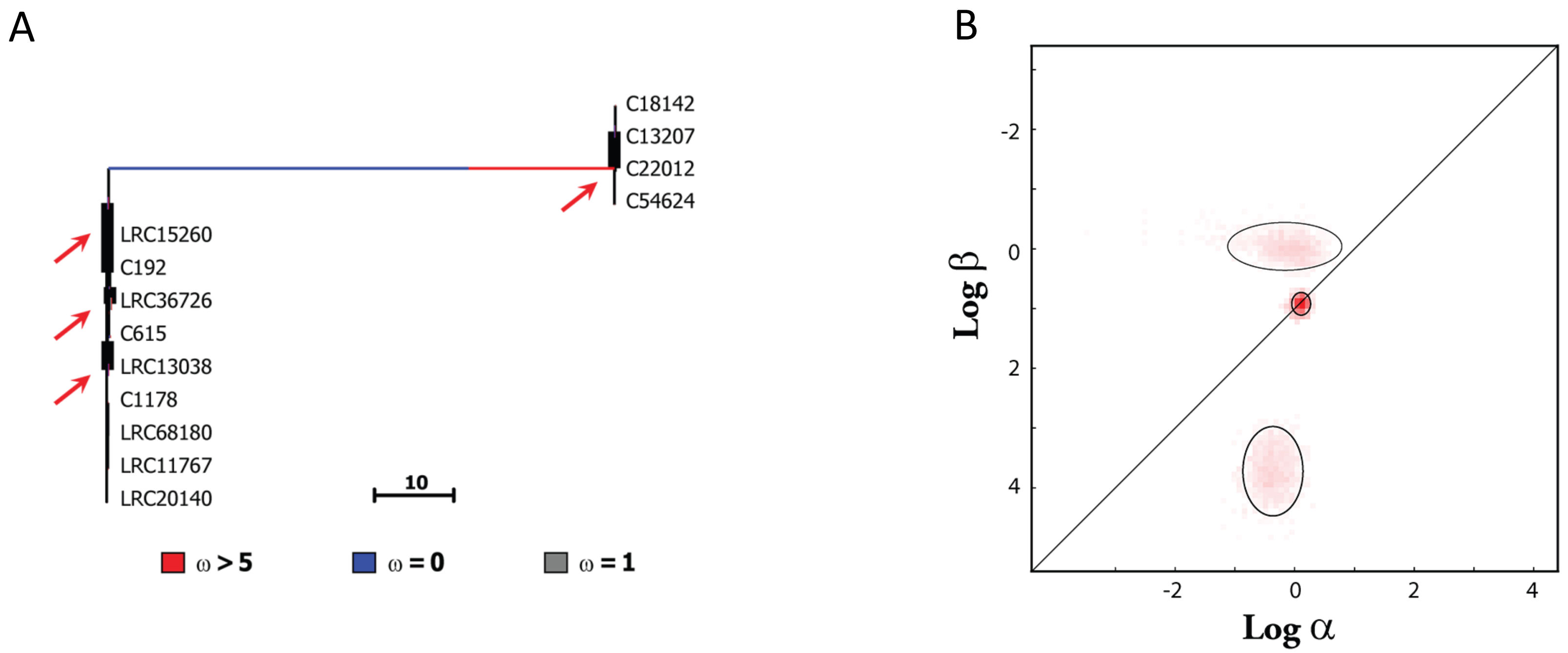

Supp. Fig. 5: Consensus sequences of top ranked & unknown putative toxins percentage of assembled reads are indicated on the left of each consensus. Red arrow indicates the cleavage site for signal peptide predicted using SignalP.

# 1
9,14 %
C--C

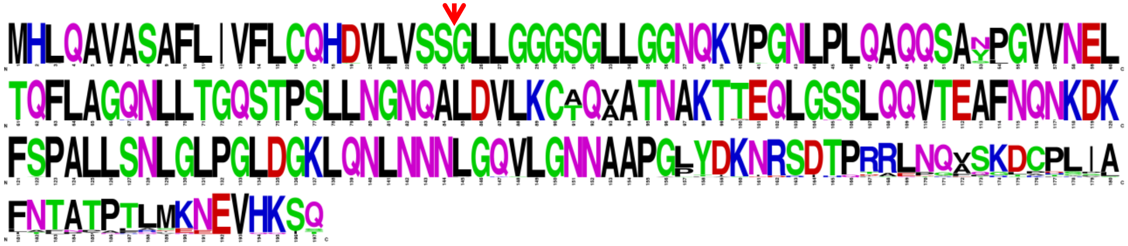

# 6
1,54 %
CC—CC--C—C—C--C

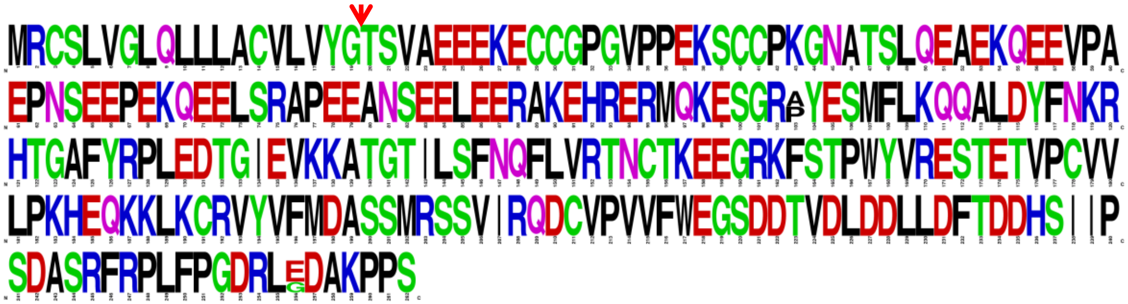

# 9
1,12 %
Cystein-free

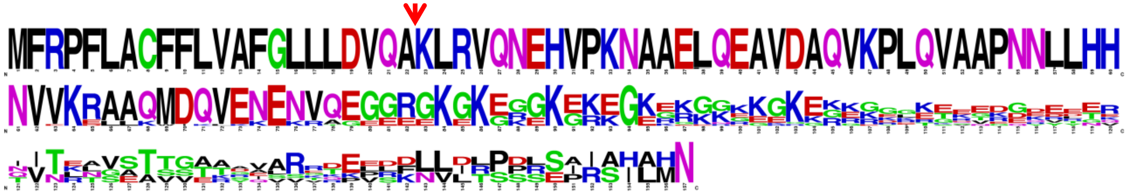

# 10
1,11 %
C—C—C—C

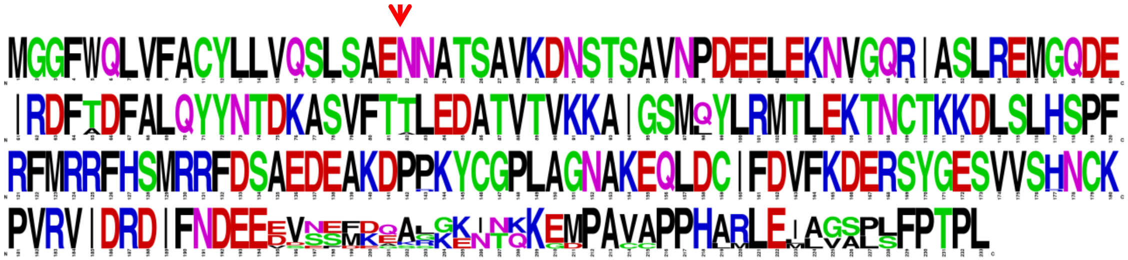

# 25
0,65 %
CCC—C—C—CC—CC—
CCC—CC—C—C—C—C

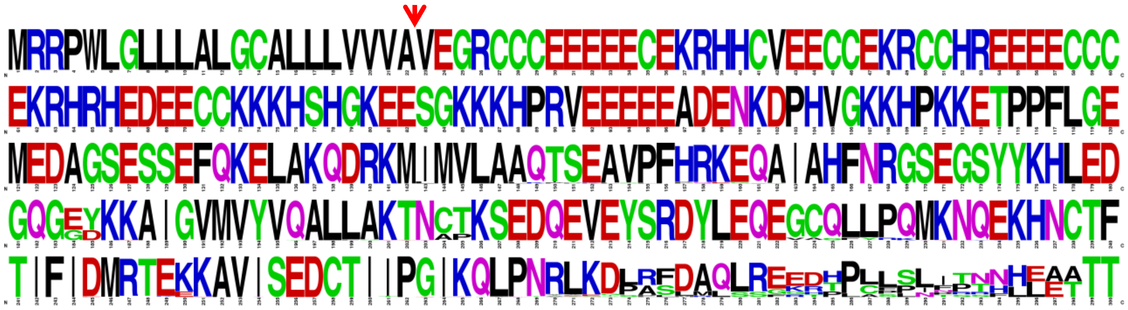

Supp. Fig. 6: Maximum likelihood tree of representative new Lipocalin toxins & consensus of group 1 & 2 isoforms

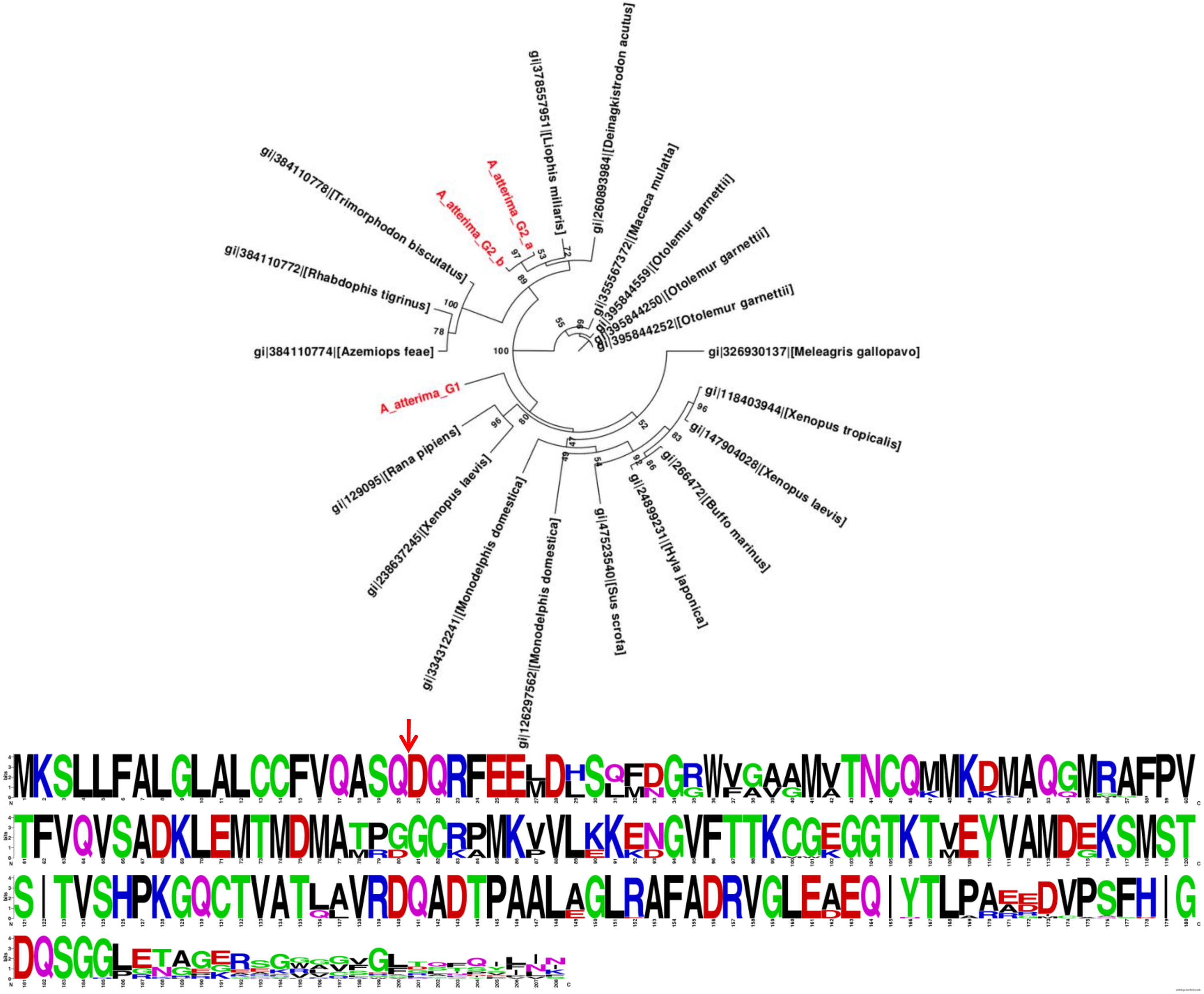

Supp. Table 1: Dynamic molecular evolution of Atractaspis 3FTx

a: Fast Unconstrained Bayesian AppRoximation

b: Sites detected as experiencing episodic diversifying selection (0.05 significance) by the Mixed Effects Model Evolution (MEME)

c: Number of branches detected as experiencing episodic diversifying selection (0.05 significance) by the Branch-site REL (Random-effects likelihood)

**FUBAR** [a]	**MEME Sites** [b]	**BSR** [c]	**PAML**		
				M8	M2a
$\omega>1$[d]: 5<br>$\omega<1$[e]: 6	8	4	$\omega>1$[f]:	17 (12+5)	15 (8+7)
			ω:	1.75	1.80
